# Immunomodulators, associated or not with systemic antibiotics, to treat periodontitis: A 1‐year multicenter, placebo‐controlled, double‐blind, randomized clinical trial

**DOI:** 10.1002/jper.70081

**Published:** 2026-05-12

**Authors:** Nidia C. Castro dos Santos, Rafael N. de Brito Silva, Fernanda S. Colombo, Ingrid Oliveira‐Cardoso, Lina J. Súarez, Rodrigo Martins, Luciene C. de Figueiredo, Heitor Marques Honório, Ademir Melo Leite Filho, Flavia A. C. Furlaneto, Michel R. Messora, Monique Furukawa, José Roberto Cortelli, Emanuel Silva Rovai, Magda Feres

**Affiliations:** ^1^ Faculdade Israelita de Ciências da Saúde Albert Einstein Hospital Israelita Albert Einstein São Paulo São Paulo Brazil; ^2^ Dental Research Division Guarulhos University Guarulhos São Paulo Brazil; ^3^ Departamento de Ciencias Básicas y Medicina Oral Universidad Nacional de Colombia Bogotá Colombia; ^4^ Centro de Investigaciones Odontológicas, Pontificia Universidad Javeriana Bogotá Colombia; ^5^ Bauru School of Dentistry University of São Paulo Bauru São Paulo Brazil; ^6^ School of Dentistry of Ribeirão Preto University of São Paulo Ribeirão Preto São Paulo Brazil; ^7^ Department of Dentistry Periodontics Research Division University of Taubaté Taubaté São Paulo Brazil; ^8^ Division of Periodontics Institute of Science and Technology São Paulo State University (Unesp) São José dos Campos São Paulo Brazil; ^9^ Department of Oral Medicine Infection, and Immunity, Harvard School of Dental Medicine, Harvard Medical School Boston Massachusetts USA

**Keywords:** antimicrobials, clinical outcomes, clinical study, omega‐3, periodontal disease, periodontal treatment, treatment planning

## Abstract

**Background:**

Immunomodulatory agents such as omega‐3 (ω‐3) and low‐dose aspirin (ASA) have been proposed as effective adjuncts in the treatment of periodontitis. However, they still lack long‐term studies and head‐to‐head evaluation with metronidazole (MTZ) and amoxicillin (AMX), the adjunct protocol with the strongest scientific evidence of clinical benefit. This study aimed to evaluate the clinical effects of (ω)‐3 and ASA, alone or combined with MTZ + AMX, to treat periodontitis.

**Methods:**

Adults with stage III/IV periodontitis were randomly allocated to receive: (i) subgingival instrumentation (SI) and placebo; (ii) SI with adjunctive MTZ (400 mg) + AMX (500 mg) three times/day for 14 days (ATB); (iii) SI with adjunctive ω‐3 (3 g) + ASA (100 mg/day) for 6 months (IM); and (iv) SI with adjunctive ATB and IM (ATB + IM). The patients were followed for 1 year post‐therapy.

**Results:**

109 patients were included. At 1 year, 58.6%, 57.7%, and 57.1% of patients receiving ATB, IM, and ATB+IM, respectively, achieved the treatment endpoint [≤4 sites with a probing depth (PD) of ≥5 mm], compared with 23.1% in the SI group (*p* = 0.023). Each adjunctive therapy resulted in a greater reduction in the number of sites with PD≥5 mm compared with SI (*p* < 0.05).

**Conclusion:**

Adjunctive ATB, IM, or ATB+IM each provided clinically meaningful benefits beyond mechanical therapy. The results suggested no added benefit from combining ATB and IM. These findings support MTZ + AMX or ω‐3 + ASA, without routine combination, as effective adjuncts in periodontal treatment and provide a basis for precision‐based trials and data‐driven models to identify patients most likely to benefit from each approach. ensaiosclinicos.gov.br RBR‐7nh566t

**Plain language summary:**

This study explored whether adding antibiotics or anti‐inflammatory supplements (omega‐3 and low‐dose aspirin), either alone or in combination, could enhance the results of subgingival instrumentation (non‐surgical periodontal therapy) in patients with severe periodontitis. All participants received the standard periodontal treatment and were randomly assigned to also take a placebo, antibiotics, omega‐3 with aspirin, or a combination of both therapies. After 1 year, patients who received any of the additional therapies showed greater improvements, with fewer sites of deep periodontal pockets, compared with those who received subgingival instrumentation alone. However, combining antibiotics with omega‐3 and aspirin did not lead to better outcomes than using either one of them individually. These findings suggest that in patients with more severe forms of periodontitis, adding either antibiotics or omega‐3 with low‐dose aspirin to standard care can provide meaningful clinical benefits.

## 1 INTRODUCTION

Periodontitis is a chronic inflammatory disease associated with a dysbiotic biofilm that destroys the periodontal supporting tissues and eventually leads to tooth loss.^1^, ^2^ The standard‐of‐care treatment for periodontitis primarily involves promoting behavior changes to enhance self‐performed supragingival biofilm control, professional mechanical biofilm removal, management of risk factors, and mechanical subgingival instrumentation (SI).^3^ Although mechanical treatment yields favorable results in reducing disease burden, it may not consistently reach optimal clinical outcomes.^4^, ^5^ Therefore, adjunctive therapies have been proposed to reduce biofilm dysbiosis or to modulate host response in individuals with severe periodontitis.

Randomized clinical trials (RCTs) suggest that the most promising adjunctive systemic antibiotic (ATB) protocol to SI is the combination of metronidazole (MTZ) and amoxicillin (AMX),^6^, ^7^, ^8^, ^9^, ^10^, ^11^ which is supported by systematic reviews and meta‐analyses.^12^, ^13^ The use of mechanical treatment combined with MTZ + AMX results in a greater percentage of patients achieving the endpoint for periodontal treatment (≤4 sites with PD ≥ 5 mm)^5^ and a lower number of residual periodontal pockets (with probing depth [PD] ≥ 5 mm) compared with SI up to 1 year post‐therapy.^7^, ^8^, ^10^, ^11^ These clinical improvements are accompanied by beneficial microbiological changes in patients receiving antibiotics.^14^, ^15^, ^16^ However, about one‐third of patients still do not achieve the clinical endpoint for periodontal treatment^5^ when using adjunctive MTZ + AMX therapy. ^6^, ^7^, ^9^, ^11^, ^16^, ^17^ Moreover, antibiotic use presents drawbacks such as potential adverse events and the risk of bacterial resistance. Therefore, other adjunctive strategies beyond MTZ + AMX should continue to be investigated.

Current evidence suggests that high‐dose omega (ω)‐3 supplementation, especially when combined with low‐dose aspirin (ASA), may improve periodontal clinical outcomes by promoting clinical attachment gain and reducing the pocket probing depth (PD).^18^, ^19^, ^20^, ^21^, ^22^, ^23^, ^24^ Additionally, omega‐3 supplementation may help to reduce pro‐inflammatory cytokines and RANKL in saliva, gingival crevicular fluid, and serum at 3 and 6 months after periodontal treatment compared with placebo groups.^25^ Nevertheless, the adjunctive benefits of omega‐3 in periodontal therapy require confirmation through large‐scale, long‐term RCTs.

Ultimately, combining antibiotics and immune modulators holds potential for improving patient outcomes. Antibiotics mitigate dysbiosis and microbial‐driven tissue damage, while host modulators promote the resolution of inflammation and tissue repair. However, their direct comparison, used alone or in combination, has never been assessed through a double‐blind, placebo‐controlled RCT. This study, therefore, aimed to evaluate the clinical effects of IM (ω‐3 + ASA), alone or combined with ATB (MTZ + AMX), compared with placebo, in patients with stage III and IV, grade B and C periodontitis.

## 2 MATERIALS AND METHODS

This study was designed as a multicenter, placebo‐controlled, double‐blind RCT with a follow‐up of 1 year to compare the use of IM, with or without ATB, to treat periodontitis. The study protocol was registered at www.ensaiosclinicos.gov.br (Trial Registration: ReBEC RBR‐7nh566t; registered August 6, 2021). The study was conducted following the Declaration of Helsinki of 1975, as revised in 2013, and approved by the Ethics Committee for research with humans from Guarulhos University, University of Taubaté, and University of São Paulo (CAAE 52333721.2.1001.5506). All study participants provided written informed consent before enrollment. The article was prepared according to the CONSORT guidelines for quality reporting RCTs.^26^

### 2.1 Study population

Patients presenting with periodontitis stage III and IV, grade B and C, were enrolled between February 2022 and February 2024 to participate in this RCT. Patients were selected at Guarulhos University, the University of Taubaté, and the University of São Paulo according to inclusion and exclusion criteria.

The inclusion criteria were periodontitis stage III or IV, grade B or C; at least 15 teeth; at least six sites with PD and CAL ≥ 5 mm; aged ≥ 30. The exclusion criteria were pregnancy or lactation; smoking, or former smoking (≤ 5 years); having received treatment for periodontitis in the previous 6 months; continuous use of oral antiseptics, systemic antibiotics, corticosteroids, non‐steroidal anti‐inflammatory medications, immunosuppressants, estrogen, and selective estrogen receptor modulators, as well as medications that may influence bone metabolism (e.g., alendronate, calcitonin, and others) in the past 6 months; systemic diseases that could alter the response to periodontal treatment (e.g., diabetes) or required prophylactic medication for dental treatment (e.g., mitral valve prolapse); reported allergies to MTZ, AMX (or any other penicillin), ASA, or fish/seafood; use of orthodontic appliances; extensive prosthetic rehabilitations; blood dyscrasias; gastritis; or gastric ulcers.

### 2.2 Sample size calculation

The ideal sample size to ensure adequate statistical power for the study was calculated based on the percentage of patients achieving the endpoint for treatment, which is ≤4 sites with PD ≥ 5 mm 1 year after therapy. A sample size of 40 patients in each group would achieve 80% power to detect a difference of 30 percentage points between each group,^11^ using a significance level of 5%. Accounting for a 25% dropout rate, the total sample size was increased to 200 patients.

### 2.3 Randomization, allocation concealment, and blinding

The study coordinator (MF) generated a random computer sequence stratified by center, employing randomly permuted blocks of sizes *n* = 2, 4, and 8. The random sequence was generated using the website Sealed Envelope (Sealed Envelope Ltd. 2022. Create a blocked randomization list. [Online] available from: https://www.sealedenvelope.com/simple‐randomiser/v1/lists). Allocation concealment was implemented using sequentially numbered, opaque, sealed envelopes, ensuring that the investigator responsible for enrolling research patients remains unaware of the allocation sequence until the individual inclusion of each patient. The researchers responsible for clinical examination (RBS, ER, AML) and subgingival instrumentation (LS, FSC, AML, FF) were blinded for group allocation.

### 2.4 Interventions

Initially, all patients received periodontal clinical examination, oral hygiene instructions (OHI), supragingival scaling using an ultrasonic scaler* and Gracey curettes†. Caries lesions were removed and sealed, and hopeless teeth were extracted before the registration of periodontal parameters. Subsequently, all patients were randomly assigned to one of the following therapeutic groups:

Placebo: SI + Placebo MTZ + AMX (thrice/daily for 14 days) + Placebo ω‐3 + ASA (daily for 6 months);
ATB: SI + 400 mg MTZ + 500 mg AMX thrice/daily for 14 days + Placebo ω‐3 + ASA (daily for 6 months);
IM: SI + 3 g ω‐3 + 100 mg ASA daily for 6 months + Placebo MTZ+AMX (thrice/daily for 14 days);
ATB+IM: SI + 400 mg MTZ + 500 mg AMX thrice/daily for 14 days + 3 g ω‐3 + 100 mg ASA daily for 6 months.

Thus, all participants received the same number of capsules of either the study medication or placebo corresponding to each of the four pharmaceutical agents evaluated.

At 3 and 6 months and 1 year post‐therapy, patients were reassessed for clinical parameters. All patients received periodontal maintenance at 3, 6, and 9 months and 1 year post‐therapy, including OHI, removal of supragingival biofilm and calculus, and re‐instrumentation of periodontal pockets under local anesthesia.

### 2.5 Clinical outcomes

The following clinical periodontal parameters were evaluated: the percentage of sites with supragingival biofilm (PI); the percentage of sites with bleeding on probing (BoP); probing depth (PD), defined as the distance (mm) from the free gingival margin to the most apical portion of the sulcus/periodontal pocket; and clinical attachment level (CAL), defined as the distance (mm) from the cementoenamel junction to the most apical portion of the sulcus/periodontal pocket. Measurements were taken at six sites per tooth (mesio‐buccal, buccal, disto‐buccal, mesio‐palatal/lingual, palatal/lingual, disto‐palatal/lingual), excluding third molars, using a North Carolina periodontal probe (PCPUNC‐BR 15)^†^.

### 2.6 Intra‐ and interexaminer calibration

An examiner in each center (RBS, ER, AML), not involved in patient treatment, was trained and intra‐calibrated to achieve maximum reproducibility of the measurements. An inter‐examiner agreement was tested against a gold standard examiner (NCCS). Continuous variables, such as PD and CAL, were assessed using the standard error of measurement (SEM), and categorical variables, such as BoP and PI, were evaluated in the intra‐ and inter‐examiner calibration through the Kappa test score (> 90%).

### 2.7 Treatment protocol

Subgingival instrumentation (SI) was performed by trained dentists (LS, FSC, AML, FF). The procedure was carried out over 2–4 sessions of approximately 1 h each, distributed over 14 days at most. SI was performed using Gracey curettes (standard and mini‐five) numbers 5/6, 7/8, 11/12, and 13/14 and ultrasonic scalers under local anesthesia. All pockets were instrumented until the complete clinically detectable removal of subgingival calculus and biofilm. Medications/placebos were prepared by a single pharmacy‡ and were initiated on the day of the first SI session. The study coordinator (NCCS) assigned the participants to the interventions according to the content of the randomization list.

### 2.8 Monitoring of compliance and adverse events

A study assistant monitored compliance with medication or placebo intake by texting or calling patients during the medication period. All capsules from the medication and the corresponding placebo presented the same color and size, and the number of capsules for the medication and placebo in each bottle was the same. The capsules were stored in coded opaque plastic bottles. The patients had to return the bottles at 7 and 14 days and 3 and 6 months after therapy to ensure compliance when they answered a questionnaire about self‐perceived adverse events. All the patients reported having adhered to the therapeutic regimen. Patients completed a self‐assessment questionnaire to report perceived adverse events at 7 and 14 days (post‐ATB or placebo for ATB) and 3 and 6 months (post‐IM or placebo for IM). The questionnaire included “yes/no/uncertain” responses for symptoms such as nausea, vomiting, or stomach pain; diarrhea; metallic taste; headache or dizziness; irritability or mood changes; weakness; excessive sleepiness; and a fish/seafood‐like taste.

### 2.9 Statistical analysis

The primary outcome variable was achieving the endpoint for periodontal treatment (≤ 4 sites with PD ≥ 5 mm) 1 year post‐therapy. Each clinical parameter assessed was computed per participant and within each group. Mean and standard deviations were calculated for each full‐mouth clinical parameter. The mean number of sites with PD ≤ 3, ≤ 4, ≥ 5, ≥ 6, and ≥ 7 mm was calculated separately within the PD thresholds per participant and then within each group. Normal distribution was tested using Shapiro–Wilk. Demographic data were assessed using one‐way ANOVA. Differences in sex and periodontitis Stages/Grades were analyzed using the Chi‐square test. The differences among full‐mouth variables were analyzed using the Repeated Measures Variance Test with Tukey post hoc. The differences in the mean number of sites within each threshold were analyzed using Generalized Estimating Equations with a Bonferroni post hoc test. The differences in the reduction of mean PI, BoP, PD, CAL, and the number of sites according to PD thresholds were analyzed using a Univariate General Linear Model. The number and percentage of patients who achieved or did not achieve the clinical endpoint for periodontal treatment were assessed using the Chi‐square test. The Bonferroni correction was applied for multiple comparisons between groups regarding the achievement or non‐achievement of the endpoint 1 year after therapy. A stepwise logistic regression analysis was performed to investigate the impact of predictor variables on the clinical target for treatment, with the three test groups being pooled as one binary (1) versus Placebo (0) outcome. The effect size of the adjunctive therapies versus the Placebo group for the number of shallow sites (PD ≤ 4 mm) and deep sites (PD ≥ 5 mm) was calculated according to Cohen's D and Glass’ Delta equations. Differences regarding adverse events were assessed using Fisher's exact test. The post‐hoc test applied for each pairwise comparison was Bonferroni. Data were analyzed using the intent‐to‐treat concept with the last observation carried forward. All data analyses were performed using IBM SPSS. The significance level applied was 5%.

## 3 RESULTS

### 3.1 Participants

**FIGURE 1:**
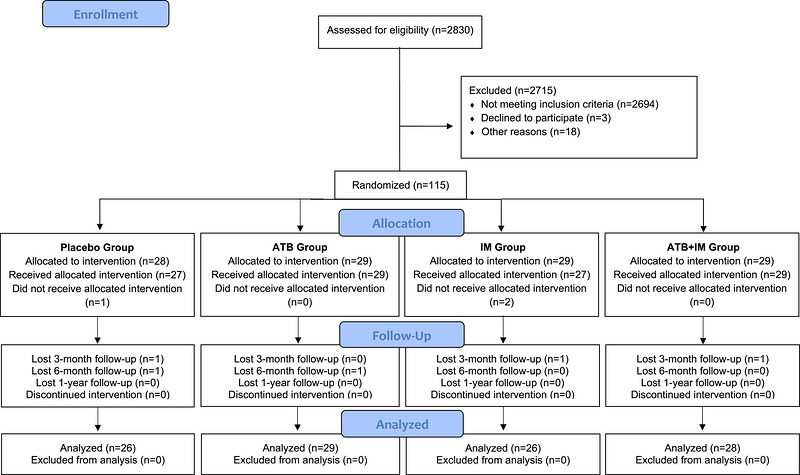
CONSORT 2010 Flow Diagram.

Figure 1 presents a CONSORT flowchart of the study. One hundred fifteen patients were selected for the study, of whom 109 received treatment and were included in the final analysis (66 females and 43 males; mean age 48.4 ± 8.5). Three participants did not receive the interventions due to schedule incompatibility (*n* = 2) and unexpected city relocation (*n* = 1); three participants were lost at the 3‐month follow‐up due to change of contact information (*n* = 2) and family reasons (*n* = 1). Table 1 shows the baseline characteristics of the study population for each group. There were no significant differences between the study groups.

**TABLE 1:** Baseline characteristics of the study population (*n* = 109).

Characteristic	Placebo group (*n* = 26)		ATB group (*n* = 29)		IM group (*n* = 26)		ATB+IM group (*n* = 28)		*p*‐value
**Sex**									
Female	16 (61.5)		18 (69.2)		18 (69.2)		15 (57.7)		>0.05
Male	10 (38.5)		11 (30.8)		8 (30.8)		13 (42.3)		
**Age (mean ± SD, years)**	47.3 ± 9.4		48.6 ± 9.7		48.1 ± 8.4		50.0 ± 6.1		>0.05
**Classification of periodontitis**									
Stage III/IV*	14 (53.8)	12 (46.2)	18 (62.1)	11 (37.9)	15 (57.7)	11 (42.3)	16 (57.1)	12 (42.9)	>0.05
Grade B/C^†^	2 (7.7)	24 (92.3)	3 (10.3)	26 (89.7)	3 (11.5)	23 (88.5)	2 (7.1)	26 (92.9)	>0.05
**Number and percentage (%) of participants according to periodontal pockets at baseline**									
6 sites with PD ≥5 mm	0		1 (3.4)		1 (3.8)		1 (3.6)		>0.05
7–8 sites with PD ≥5 mm	1 (3.8)		1 (3.4)		0		1 (3.6)		>0.05
≥9 sites with PD ≥5 mm	25 (96.2)		27 (93.1)		25 (96.2)		26 (92.9)		>0.05

*Note*: The treatment groups were compared using one‐way ANOVA and the Pearson Chi‐Square test (*p* < 0.05).

* Within each group/column, the number and percentage to the left is patients with stage III periodontitis and to the right, patients with stage IV periodontitis.

† Within each group/column, the number and percentage to the left is patients with grade B periodontitis and to the right, patients with grade C periodontitis.

### 3.2 Primary outcome

At 1 year post‐therapy, the number and percentage of patients who achieved the endpoint for treatment were 6 (23.1%) in the Placebo group, 17 (58.6%) in the ATB group, 15 (57.7%) in the IM group, and 16 (57.1%) in the ATB+IM group. Patients who received adjunctive ATB, IM, or ATB + IM showed a markedly higher response rate than those in the Placebo group. This difference was statistically significant (*p* = 0.023; Table 2). In the pairwise comparisons, a significant difference was observed between the Placebo group and each of the treatment groups (*p* < 0.001). No significant difference was found between the ATB and IM groups (*p* = 0.0282), nor between the IM and ATB+IM groups (*p* = 0.2000). However, a significant difference was detected between the ATB and ATB+IM groups (*p* = 0.0004) (Table 2).

**TABLE 2:** The number and percentage of patients who achieved or did not achieve the endpoint for periodontal treatment (≤ 4 sites with PD ≥ 5 mm) for the Placebo, ATB, IM, and ATB+IM Groups 1 year post‐treatment (*n* = 109 patients).

Achieved the endpoint for treatment	Placebo group (*n* = 26)	ATB group (*n* = 29)	IM group (*n* = 26)	ATB+IM group (*n* = 28)	*p*‐value
3 months	6 (23.1%)^A^	6 (20.7%)^A^	6 (23.1%)^A^	7 (25%)^A^	0.985
6 months	7 (26.9%)^A^	15 (51.7%)^A^	15 (57.7%)^A^	14 (50%)^A^	0.124
1 year	6 (23.1%)^A^	17 (58.6%)^B^	15 (57.7%)^B^	16 (57.1%)^B^	**0.023**

**Comparisons between groups at 1 year**	**Placebo group** **(*n* = 26)**	**ATB group** **(*n* = 29)**	**IM group** **(*n* = 26)**	**ATB+IM group** **(*n* = 28)**	**Bonferroni‐adjusted** ***p‐*value**
Placebo versus ATB	6 (23.1%)^A^	17 (58.6%)^B^	–	–	**<0.0001**
Placebo versus IM	6 (23.1%)^A^	–	15 (57.7%)^B^	–	**<0.0001**
Placebo versus ATB+IM	6 (23.1%)^A^	–	–	16 (57.1%)^B^	**<0.0001**

Treatment effectiveness	Absolute risk difference	Number needed to treat			
Placebo versus ATB	36%	3			
Placebo versus IM	35%	3			
Placebo versus ATB+IM	34%	3			

*Note*: The treatment groups were compared using the Pearson Chi‐Square test according to the endpoint categories (*p* < 0.05). Different superscript letters indicate statistically significant differences between groups.

Abbreviations: ATB, antibiotics; IM, immunomodulators; PD, probing depth.

We evaluated the treatment endpoint at 3 and 6 months of follow‐up. At the 3‐month assessment, no statistically significant differences were observed among the groups. However, by the 6‐month mark, this pattern had shifted. The number of patients achieving the endpoint increased substantially in the adjunctive treatment groups, while the Placebo group showed only a modest increase. At the 1‐year follow‐up, the Placebo group remained relatively stable, whereas the ATB, IM, and ATB+IM groups demonstrated sustained improvement (Table 2). These findings suggest that the adjunctive therapies provided progressive benefits over time, in contrast to the Placebo group, which appeared to plateau.

### 3.3 Secondary outcomes

The full‐mouth clinical parameters are presented in Table 3. There were significant intragroup differences in the assessed variables for the four treatment groups, with no significant differences between groups (Table 3).

**TABLE 3:** Full‐mouth periodontal clinical parameters for the Placebo, ATB, IM, and ATB+IM groups at baseline, 3 and 6 months, and 1 year post‐therapy (*n* = 109 patients).

Variable	Time point	Placebo group (*n* = 26)	ATB group (*n* = 29)	IM group (*n* = 26)	ATB+IM group (*n* = 28)	*p*‐value
PI (%)	Baseline	63.6 ± 2.8^Aa^	52.5 ± 2.6^Aa^	65.5 ± 2.2^Aa^	49.5 ± 1.7^Aa^	>0.05
	3 months	24.9 ± 1.7^Ab^	28.1 ± 1.7^Ab^	19.4 ± 1.4^Ab^	31.0 ± 1.6^Ab^	>0.05
	6 months	26.8 ± 1.5^Ab^	23.8 ± 1.5^Ab^	15.8 ± 1.2^Ab^	26.3 ± 1.7^Ab^	>0.05
	1 year	20.2 ± 1.4^Ab^	18.8 ± 1.3^Ab^	20.2 ± 1.2^Ab^	22.0 ± 1.5^Ab^	>0.05
	Δ BL‐1Y	43.4 ± 1.5^A^	33.7 ± 1.4^A^	45.3 ± 1.4^A^	27.5 ± 1.6^A^	>0.05
BoP (%)	Baseline	50.1 ± 2.4^Aa^	47.3 ± 2.4^Aa^	48.9 ± 2.5^Aa^	45.0 ± 2.0^Aa^	>0.05
	3 months	18.9 ± 1.1^Ab^	18.9 ± 1.3^Ab^	17.8 ± 1.4^Ab^	20.6 ± 1.3^Ab^	>0.05
	6 months	14.9 ± 1.1^Ab^	14.8 ± 1.2^Ab^	14.7 ± 1.3^Ab^	18.4 ± 0.9^Ab^	>0.05
	1 year	12.2 ± 1.0^Ab^	13.9 ± 1.2^Ab^	13.5 ± 1.4^Ab^	14.8 ± 1.0^Ab^	>0.05
	Δ BL‐1Y	37.9 ± 1.2^A^	33.4 ± 1.3^A^	35.4 ± 1.3^A^	30.2 ± 1.0^A^	>0.05
PD (mm)	Baseline	2.6 ± 0.6^Aa^	2.7 ± 0.6^Aa^	2.9 ± 0.8^Aa^	2.6 ± 0.5^Aa^	>0.05
	3 months	1.9 ± 0.5^Ab^	2.0 ± 0.5^Ab^	2.1 ± 0.6^Ab^	2.2 ± 0.4^Ab^	>0.05
	6 months	1.9 ± 0.5^Ab^	1.9 ± 0.5^Ab^	2.1 ± 0.4^Ab^	2.1 ± 0.4^Ab^	>0.05
	1 year	1.8 ± 0.4^Ab^	1.9 ± 0.5^Ab^	2.0 ± 0.6^Ab^	2.1 ± 0.5^Ab^	>0.05
	Δ BL‐1Y	0.8 ± 0.4^A^	0.8 ± 0.4^A^	0.9 ± 0.5^A^	0.5 ± 0.4^A^	>0.05
CAL (mm)	Baseline	3.0 ± 1.0^Aa^	3.0 ± 0.9^Aa^	3.0 ± 0.9^Aa^	2.6 ± 1.2^Aa^	>0.05
	3 months	2.3 ± 0.6^Ab^	2.3 ± 0.7^Ab^	2.4 ± 0.6^Ab^	2.2 ± 1.1^Ab^	>0.05
	6 months	2.4 ± 0.7^Ab^	2.2 ± 0.7^Ab^	2.5 ± 0.6^Ab^	2.1 ± 1.0^Ab^	>0.05
	1 year	2.2 ± 0.6^Ab^	2.2 ± 0.6^Ab^	2.4 ± 0.7^Ab^	2.0 ± 1.0^Ab^	>0.05
	Δ BL‐1Y	0.8 ± 0.6^A^	0.7 ± 0.6^A^	0.6 ± 0.7^A^	0.6 ± 0.8^A^	>0.05

*Note*: Significant differences were analyzed using Repeated Measures Analysis of Variance with Tukey *post hoc*. Different lowercase letters represent intragroup differences (*p *< 0.05). The mean changes (Δ) in the number of sites within each threshold were compared using the Univariate General Linear Model with DMS *post hoc* (*p *< 0.05).

Abbreviations: ATB, antibiotics; BoP, bleeding on probing; CAL, clinical attachment level; IM, immunomodulators; PD, probing depth; PI, plaque Index.

The number of sites within different thresholds for PD was compared over time between groups (Table 4). The mean increase in the number of sites with PD ≤ 3 mm was 11.1 ± 4.2, 23.2 ± 4.1, 28.4 ± 4.0, and 22.6 ± 4.0 for the Placebo, ATB, IM, and ATB+IM groups, respectively (*p* < .049). For the sites with PD ≤ 4 mm, the mean increase was 9.3 ± 3.0, 20.5 ± 2.8, 19.0 ± 3.0, and 20.6 ± 3.0 for the Placebo, ATB, IM, and ATB+IM groups, respectively (*p* < 0.022). The mean reduction in the number of sites with PD ≥ 5 mm and the percentage of “pocket closure” (sites changing from ≥ 5 mm to ≤ 3 mm) were 12.8 ± 2.8 (62.1%), 20.5 ± 2.6 (74.1%), 20.8 ± 2.8 (72.9%), and 18.3 ± 2.8 (70.4%) for the groups Placebo, ATB, IM, and ATB+IM, respectively (*p* < 0.048).

**TABLE 4:** Number of shallow and deep sites (mean ± SD) within different thresholds for the Placebo, ATB, IM, and ATB+IM groups at baseline, 3 and 6 months, and 1 year post‐therapy (*n* = 109 patients).

Variable	Time point	Placebo group (*n* = 26)	ATB group (*n* = 29)	IM group (*n* = 26)	ATB+IM group (*n* = 28)	*p*‐value
Sites with PD ≤ 3 mm	Baseline	105.1 ± 4.5	103.8 ± 4.8^a^	99.9 ± 6.0^a^	103.9 ± 5.2^a^	>0.05
	3 months	116.2 ± 4.7	125.3 ± 3.3^b^	117.3 ± 4.8^b^	124.4 ± 4.8^b^	>0.05
	6 months	116.1 ± 4.7	126.4 ± 3.5^b^	122.2 ± 5.0^b^	124.7 ± 4.6^b^	>0.05
	1 year	117.8 ± 4.5	126.9 ± 3.5^b^	125.7 ± 4.9^b^	126.8 ± 4.3^b^	>0.05
	Δ BL‐1Y	11.1 ± 4.2^A^	23.2 ± 4.1^B^	28.4 ± 4.0^B^	22.6 ± 4.0^A^	<0.049
Sites with PD ≤ 4 mm	Baseline	119.3 ± 4.2	121.0 ± 4.3^a^	116.5 ± 5.3	122.2 ± 4.7^a^	>0.05
	3 months	125.6 ± 4.5	137.1 ± 3.0^b^	129.7 ± 4.2^b^	138.5 ± 3.9^b^	>0.05
	6 months	126.3 ± 4.2	137.5 ± 3.2^b^	133.1 ± 4.0^b^	138.1 ± 3.8^b^	>0.05
	1 year	126.5 ± 4.5	140.0 ± 2.6^b^	135.0 ± 4.1^b^	141.2 ± 3.4^b^	>0.05
	Δ BL‐1Y	9.3 ± 3.0^A^	20.5 ± 2.8^B^	19.0 ± 3.0^B^	20.6 ± 3.0^B^	<0.022
Sites with PD ≥ 5 mm	Baseline	21.1 ± 2.2^a^	25.5 ± 3.0^a^	28.0 ± 3.4^a^	25.3 ± 2.9^a^	>0.05
	3 months	9.0 ± 1.1^b^	9.3 ± 1.0^b^	10.2 ± 1.1^b^	9.3 ± 0.9^b^	>0.05
	6 months	8.4 ± 1.1^b^	7.8 ± 1.0^b^	9.2 ± 1.4^b^	9.9 ± 1.3^b^	>0.05
	1 year	8.0 ± 1.1^b^	6.6 ± 1.2^b^	7.6 ± 1.3^b^	7.5 ± 0.9^b^	>0.05
	Δ BL‐1Y	12.8 ± 2.8^A^	20.5 ± 2.6^B^	20.8 ± 2.8^B^	18.3 ± 2.8^B^	<0.048
Sites with PD ≥ 6 mm	Baseline	11.6 ± 1.9^a^	14.6 ± 2.4^a^	17.0 ± 2.9^a^	15.2 ± 2.3^a^	>0.05
	3 months	5.0 ± 0.7	4.2 ± 0.5^b^	6.6 ± 0.8^a^	5.5 ± 0.8^b^	>0.05
	6 months	4.5 ± 0.7^b^	4.1 ± 0.9^b^	5.6 ± 1.0^b^	4.6 ± 0.5^b^	>0.05
	1 year	3.7 ± 0.6^b^	3.5 ± 1.0^b^	4.8 ± 0.7^b^	4.2 ± 0.6 ^b^	>0.05
	Δ BL‐1Y	7.9 ± 2.4	12.3 ± 2.3	12.8 ± 2.4	11.9 ± 2.4	>0.05
Sites with PD ≥ 7 mm	Baseline	6.7 ± 1.3^a^	7.0 ± 1.4^a^	9.9 ± 2.1^a^	7.9 ± 1.5^a^	>0.05
	3 months	2.6 ± 0.3^b^	2.2 ± 0.4^b^	3.3 ± 0.6^b^	3.2 ± 0.6^b^	>0.05
	6 months	2.4 ± 0.4^b^	2.3 ± 0.5^b^	3.2 ± 0.7^b^	2.6 ± 0.4^b^	>0.05
	1 year	2.3 ± 0.4^b^	2.2 ± 0.7^b^	2.6 ± 0.4^b^	2.7 ± 0.5^b^	>0.05
	Δ BL‐1Y	3.9 ± 1.4	5.6 ± 1.3	5.6 ± 1.4	5.8 ± 1.4	>0.05

*Note*: The mean number of sites within each threshold was analyzed using Generalized Estimating Equations with Bonferroni *post hoc*. Different lowercase letters represent intragroup differences (*p *< 0.05). The mean changes (Δ) in the number of sites within each threshold were compared using the Univariate General Linear Model with DMS *post hoc* (*p *< 0.05).

Abbreviations: ATB, antibiotics; IM, immunomodulators; PD, probing depth.

### 3.4 Exploratory outcomes

Logistic regression analysis showed that mean PI, mean PD, and using adjunctive therapy (OR = 3.5) influenced achieving or not achieving the treatment endpoint 1‐year post‐treatment (*p* = 0.005, *p* = 0.006, and *p* = 0.019, respectively; Table 5). Multicollinearity was tested prior to model fitting using variance inflation factors (VIF), and no significant collinearity was observed among the predictors.

**TABLE 5:** Binary logistic regression considering achieving the endpoint for periodontal treatment (≤ 4 sites with PD ≥ 5 mm) 1 year post‐treatment as the dependent outcome variable.

Variables that influenced the response to treatment	*p‐*value	OR	95% CI
PI	0.005	15.6	2.3–106.7
PD	0.006	0.4	0.2–0.8
Adjunctive therapy	0.019	3.3	1.2–8.7

*Note*: Independent variables included PI (%), BoP (%), mean PD (mm), mean CAL (mm), age, and the use of adjunctive therapy (*n* = 109). Nagelkerke *R* ^2^ = 0.182; *p *= 0.016.

Abbreviations: CI, confidence interval; PD, probing depth; PI, plaque index; CAL, clinical attachment level.

The effect size (ES) of each adjunctive therapy versus Placebo on the increase in the number of shallow sites (PD ≤ 4 mm) and the reduction of deep sites (PD ≥ 5 mm) is shown in Table S1 in the online *Journal of Periodontology*. For the increase in the number of sites with PD ≤ 4 mm, the ES was −0.57, −0.63, and −0.50 (Cohen's D) for the ATB versus Placebo, IM versus Placebo, and ATB+IM versus Placebo, respectively. For the reduction in the number of sites with PD ≥ 5 mm, the ES was 0.96, 0.99, and 0.46 (Glass’ Delta) for the ATB versus Placebo, IM versus Placebo, and ATB+IM versus Placebo, respectively. For patients presenting with ≥ 9 sites with PD ≥ 5 mm at baseline, the ES (Cohen's D) for the increase in the number of sites with PD ≤ 4 mm were −0.63, −1.70, and −1.30 for the ATB versus Placebo, IM versus Placebo, and ATB+IM versus Placebo, respectively. For the reduction in the number of sites with PD ≥ 5 mm, the ES (Glass’ Δ) was 0.68, 0.80, and 0.55 for the ATB versus Placebo, IM versus Placebo, and ATB+IM versus Placebo, respectively.

### 3.5 Adverse events

Table S2 in the online *Journal of Periodontology* shows the number and frequency of patients’ responses for self‐perceived symptoms related to adverse events at 7 and 14 days (post ATB or placebo), and 3 and 6 months (post‐IM or placebo). No significant differences were observed between the groups at any of the four follow‐up time points. Nonetheless, there was a non‐significant trend indicating that a higher percentage of patients in the IM group reported a fish/seafood‐like taste compared with the other groups, with no statistical significance (*p* > 0.05). Importantly, no severe adverse events requiring medical intervention or hospitalization were reported throughout the study.

## 4 DISCUSSION

To the best of our knowledge, this is the first double‐blind, placebo‐controlled RCT to directly evaluate IM, MTZ + AMX, and their combination as adjuncts to mechanical debridement, relative to mechanical therapy alone. Although the study was not powered for comparisons between adjunctive regimens, it provides important preliminary evidence on their comparative performance in the treatment of periodontitis. A significantly higher percentage of patients in the IM (57.7%), ATB (58.6%), and ATB+IM (57.1%) groups achieved the clinical endpoint for treatment (primary outcome) compared with the Placebo group (23.1%) (*p* = 0.023). In the multiple comparisons between groups, the percentage of individuals achieving the treatment endpoint was significantly higher when comparing ATB versus Placebo, IM versus Placebo, and ATB+IM versus Placebo (adjusted *p* < 0.001) (Table 2).

These findings for the primary outcome variable are consistent with previous studies on the effects of MTZ + AMX, reinforcing its role as an effective adjunctive therapy in periodontal treatment.^7^, ^9^, ^10^, ^17^ Regarding the antibiotic protocol used in this study, it is important to highlight that the 14‐day protocol of 400 mg MTZ + 500 mg AMX was adopted in prior RCTs ^6‐9,11^, demonstrating superior clinical outcomes with this duration, in comparison with mechanical treatment. In particular, the only RCT trial directly comparing 7‐ versus 14‐day regimens against placebo as adjuncts to mechanical treatment showed that the 14‐day treatment achieved higher rates of successful periodontal therapy.^11^

Direct comparison with studies assessing the adjunctive use of ω‐3 fatty acids combined with ASA is more limited, as most did not report this specific clinical endpoint.^20^, ^22^, ^24^, ^27^ One study, however, found that 40% of patients with type 2 diabetes who received 3 g ω‐3 + 100 mg ASA for 2 months achieved the treatment endpoint (≤4 sites with PD ≥ 5 mm) at 6 months post‐therapy.^18^ In contrast, 57.7% of patients in the present study reached this outcome. These differences may reflect variations in therapeutic protocols, as Castro dos Santos (2020) administered ω‐3 + ASA for only 2 months, whereas the current trial extended the treatment to 6 months. Furthermore, the effects of ω‐3 + ASA may differ between patients with uncontrolled diabetes^18^ and those with normoglycemia, potentially amplifying the benefits observed in the current study's population.

As secondary outcomes, we evaluated the number of sites within various probing depth (PD) thresholds as secondary outcomes of the study. The adjunctive therapies (ATB, IM, and ATB+IM) resulted in significantly greater increases in the number of shallow sites (PD ≤ 3 mm and ≤ 4 mm) compared with the Placebo group. Similarly, these groups demonstrated a greater reduction in the number of sites with PD ≥ 5 mm relative to the Placebo group. Notably, intra‐group comparisons in the Placebo group revealed no significant changes in the number of sites with PD ≤ 3 mm or ≤ 4 mm, suggesting that SI alone was insufficient to increase the number of healthy sites over the study period. For deeper periodontal pockets (PD ≥ 6 mm and ≥ 7 mm), adjunctive therapies tended to yield fewer sites at the 1‐year follow‐up and showed a greater reduction in the number of sites between baseline and 1 year. However, these differences were not statistically significant.

We also conducted an exploratory analysis to evaluate the ES of each adjunctive therapy compared with the Placebo group regarding the increase in the number of shallow sites (PD ≤ 4 mm) and the reduction of the number of deep sites (PD ≥ 5 mm) from baseline to 1 year. For the increase in shallow sites, the adjunctive therapies (ATB, IM, and ATB+IM) demonstrated a medium effect size relative to the Placebo group. Regarding reducing the number of deep sites, ATB and IM exhibited a large effect size, whereas ATB+IM showed a small ES compared with Placebo.^28^ These findings suggest that the use of ATB or IM individually may offer greater benefits in reducing the number of deep periodontal pockets compared with their combined use (ATB+IM). In a subset analysis of patients presenting the highest number of sites with PD ≥ 5 mm at baseline (with ≥ 9 sites), the IM treatment showed a greater effect size in reducing the number of these deep sites compared with the ATB and the ATB+IM groups, suggesting that host‐modulating agents may be more effective than MTZ+AMX in reducing the number of deep pockets in patients with increased disease severity at baseline. However, these findings would have to be confirmed by future RCTs powered to compare directly these treatment protocols.

Overall, the results suggested that combining ATB and IM may not provide additional clinical benefits over using them individually.

The absence of an additive benefit when combining systemic ATB with IM may, at least in part, reflect a ceiling effect within this population. Each therapy targets a distinct component of the disease. Antibiotics suppress dysbiosis and microbial‐driven tissue damage, whereas immunomodulators promote inflammation resolution and tissue repair, but both ultimately converge on restoring periodontal homeostasis. It is therefore plausible that, once one pathway adequately controls the infection–inflammation cycle, the scope for further clinical improvement through the other becomes limited. This hypothesis may be particularly relevant for patients with moderate‐to‐severe periodontitis and generally good systemic health, as in the present cohort, where a ceiling effect is more likely to occur. Temporal dynamics may also contribute, since most adjunctive benefits from either therapy arise within the first 3–6 months following SI,^8^, ^11^, ^18^, ^20^ reducing the opportunity for cumulative gains when both are applied simultaneously. Altogether, these observations suggest that the absence of an additive effect does not imply therapeutic antagonism, but rather that each intervention alone may approach the maximal clinical response achievable in this setting. From a clinical perspective, avoiding unnecessary combination therapy may also confer economic benefits and improve patient acceptability, as single‐modality adjuncts reduce complexity and cost.

The exploratory stepwise binary logistic regression identified baseline PI, baseline PD, and adjunctive therapy as significant predictors of achieving the periodontal treatment endpoint at 1 year. These results indicate that the adjunctive therapies significantly increase the likelihood of achieving the endpoint (OR = 3.5) and further emphasize that effective plaque control remains a key determinant of treatment success, underscoring the importance of biofilm control during the active phase of treatment.

It is important to note that achieving the clinical endpoint of ≤4 sites with PD ≥5 mm is not merely a surrogate metric of short‐term therapeutic success, but a meaningful indicator of long‐term periodontal stability and tooth preservation. A recent systematic review and meta‐analysis, including 12,884 patients in supportive periodontal care, demonstrated that failure to achieve this endpoint was associated with a 2.6‐fold higher risk of tooth loss (RR = 2.57).^29^ Based on these findings and the results of the present study, it is reasonable to conclude that the adjunctive therapies evaluated here may reduce the risk of tooth loss by increasing the likelihood of patients achieving controlled periodontitis. This is clinically relevant because preventing tooth loss remains a central goal of periodontal therapy and has far‐reaching implications for function, esthetics, nutrition, and overall quality of life.^30^, ^31^ Moreover, tooth loss has been increasingly linked to adverse systemic outcomes, including frailty, cognitive decline, cardiovascular disease, and reduced longevity.^32^, ^33^, ^34^, ^35^ From a therapeutic standpoint, these findings reinforce the evidence supporting ATB and IM strategies as effective approaches to periodontal treatment.

The most notable finding in this study was that the ω‐3 + ASA protocol achieved clinical outcomes comparable to those of the ATB regimen when each was compared with Placebo, while remaining well‐tolerated over the 6 months. These results are particularly relevant in light of the global concern over antimicrobial resistance, emphasizing the potential of host‐modulating agents as a safe and effective alternative to systemic antibiotics. Importantly, the findings apply primarily to systemically healthy patients with stages III/IV generalized periodontitis but a lower extent of disease (21–28 mean number of sites with PD ≥ 5 mm), compared with earlier antibiotic trials (32–60 mean number of sites with PD ≥ 5 mm). Together, these findings provide novel and clinically meaningful evidence supporting both ATB and IM agents as valuable adjuncts in distinct clinical contexts. Antibiotics showed consistent effectiveness even for patients with lower disease extent, while host modulators offered comparable benefits with an improved safety profile. Future studies integrating phenotypically diverse populations will be essential to identify patient subgroups most likely to benefit from each adjunctive strategy.

This study had limitations. First, the smaller‐than‐planned sample size represents a key drawback. Recruitment began in early 2022, and the lingering effects of the COVID‐19 pandemic in Brazil, together with budgetary and logistical constraints, limited enrollment. Although the study was not powered for direct comparisons between the three test groups, it was prospectively powered to compare each adjunctive therapy with placebo. With 26–29 patients per group, the achieved power (≈75%) was slightly below the planned 80%, yet sufficient to support the main clinical conclusions based on the 34‐percentage‐point difference observed between groups.

In addition, although all participants were classified as having stage III or IV, grade B or C periodontitis, we were unable to distinguish between these categories, and variability within them may have influenced the outcomes. Moreover, the study sample consisted exclusively of non‐smoking individuals without systemic conditions; therefore, the results may not be directly generalizable to patients with systemic risk factors, who could respond differently to adjunctive therapies. Also, this study tested the effectiveness of IM and ATB, not efficiency, as the cost‐benefit ratio has not been assessed. Finally, the 1‐year follow‐up corresponds to 6 months after the completion of IM administration but 1 year after MTZ+AMX administration, which may have favored the outcomes observed in the IM group.

This study's primary strength lies in its pioneering design as the first randomized, double‐blind, placebo‐controlled clinical trial to evaluate the long‐term (1 year) effects of adjunctive ω‐3 and ASA therapy, and to assess, under identical experimental conditions, three distinct adjunctive strategies—MTZ+AMX, (ω)‐3 and ASA, and their combination—against SI alone with placebo. Conducting all treatment arms within a single, rigorously controlled study allowed for a unified evaluation of outcomes while eliminating the variability inherent to cross‐trial comparisons. By including more than 100 patients across three clinical centers in different cities, the study also enhances external validity and generalizability.

## 5 CONCLUSION

In conclusion, in stage III/IV periodontitis, adjunctive ATB, IM, or ATB+IM each conferred clinically meaningful benefits beyond mechanical therapy alone. However, the results suggested no added benefit from combining ATB and IM. These findings support MTZ+AMX or ω‐3+ASA, without routine combination, as effective adjuncts in periodontal treatment. These findings provide a foundation for precision trials and data‐driven models to identify patients most likely to benefit from each approach. Future studies including phenotypically diverse populations, extended follow‐up, and adequate power to compare between adjunctive therapies—while integrating microbiological and immunological analyses—will be important to determine whether specific patient subgroups benefit preferentially from one approach and to further elucidate the biological mechanisms underlying these effects.

## AUTHOR CONTRIBUTIONS

**Nidia C. Castro dos Santos**: Funding; coordinator; research protocol development; data interpretation; first and final draft. **Rafael N. de Brito Silva**: Examiner; final draft. **Fernanda S. Colombo**: Treatment of patients; final draft. **Ingrid Oliveira‐Cardoso**: Treatment of patients; final draft. **Lina J. Suarez**: Treatment of patients; final draft. **Rodrigo Martins**: Treatment of patients; final draft. **Luciene de Figueiredo**: Data interpretation; final draft. **Heitor Marques Honório**: Statistical analysis; data interpretation; final draft. **Ademir Melo Leite Filho**: Examiner; final draft. **Flavia Furlaneto**: Treatment of patients; final draft. **Michel Messora**: Clinical center coordinator; final draft. **Monique Furukawa**: Treatment of patients; final draft. **José Roberto Cortelli**: Treatment of patients; final draft. **Emanuel da Silva Rovai**: Clinical center coordinator; examiner; final draft. **Magda Feres**: Principal investigator; funding; research protocol development; data interpretation; second and final draft.

## CONFLICT OF INTEREST STATEMENT

The authors have no conflicts of interest to disclose.

## Supporting information



Supporting Information

Supporting Information

## Footnotes

^*^
Dabi Atlante, Ribeirao Preto, SP, Brazil

^†^
Hu‐Friedy, Chicago, IL, USA

^‡^
Farmácia Terapêutica, São José dos Campos, SP, Brazil
