# Supplementary material for: Immunomodulators, associated or not with systemic antibiotics, to treat periodontitis: A 1‐year multicenter, placebo‐controlled, double‐blind, randomized clinical trial
Source: J Periodontol. 2026 May 12;97(7):1454–66. doi: 10.1002/jper.70081 (PMC13380354; doi:10.1002/jper.70081)
Supplement: Supplementary file 1 — Supporting Information [file JPER-97-1454-s002.docx]

|  | Time point | Placebo Group  (n=17) | ATB Group  (n=17) | IM Group  (n=18) | ATB+IM Group (n=17) | p value |
| --- | --- | --- | --- | --- | --- | --- |
| Nausea, vomiting or stomach pain | 7 days  14 days  3 months  6 months | 2 (11.8)  0 (0)  2 (11.8)  1 (5.9) | 4 (23.5)  3 (17.6)  1 (5.9)  0 (0) | 4 (22.2)  2 (11.1)  4 (22.2)  2 (11.1) | 2 (11.8)  0 (0)  2 (11.8)  2 (11.8) | > .05  > .05  > .05  > .05 |
| Diarrhea | 7 days  14 days  3 months  6 months | 1 (5.9)  0 (0)  2 (11.8)  0 (0) | 1 (5.9)  1 (5.9)  2 (11.8)  0 (0) | 2 (11.1)  1 (5.6)  3 (16.7)  1 (5.6) | 3 (17.6)  0 (0)  0 (0)  0 (0) | > .05  > .05  > .05  > .05 |
| Metallic taste | 7 days  14 days  3 months  6 months | 2 (11.8)  2 (11.8)  1 (5.9)  0 (0) | 1 (5.9)  0 (0)  1 (5.9)  2 (11.8) | 3 (16.7)  1 (5.6)  2 (11.1)  3 (16.7) | 3 (17.6)  0 (0)  2 (11.8)  1 (5.9) | > .05  > .05  > .05  > .05 |
| Headache or dizziness | 7 days  14 days  3 months  6 months | 4 (23.5)  3 (16.7)  2 (11.8)  4 (23.5) | 3 (17.6)  0 (0)  3 (17.6)  1 (5.9) | 5 (27.8)  4 (22.2)  6 (33.3)  5 (27.8) | 3 (17.6)  2 (11.8)  3 (17.6)  1 (5.9) | > .05  > .05  > .05  > .05 |
| Irritability or mood changes | 7 days  14 days  3 months  6 months | 1 (5.9)  1 (5.9)  0 (0)  0 (0) | 1 (5.9)  1 (5.9)  1 (5.9)  0 (0) | 0 (0)  0 (0)  1 (5.6)  1 (5.6) | 0 (0)  0 (0)  1 (5.9)  0 (0) | > .05  > .05  > .05  > .05 |
| Weakness | 7 days  14 days  3 months  6 months | 1 (5.9)  0 (0)  0 (0)  1 (5.9) | 3 (17.6)  1 (5.9)  1 (5.9)  0 (0) | 4 (22.2)  2 (11.1)  1 (5.6)  2 (11.1) | 1 (5.9)  0 (0)  0 (0)  0 (0) | > .05  > .05  > .05  > .05 |
| Excessive sleepiness | 7 days  14 days  3 months  6 months | 1 (5.9)  1 (5.9)  1 (5.9)  1 (5.9) | 3 (17.6)  2 (11.8)  4 (23.5)  1 (5.9) | 4 (22.2)  3 (16.7)  4 (22.2)  2 (11.1) | 1 (5.9)  2 (11.8)  2 (11.8)  1 (5.9) | > .05  > .05  > .05  > .05 |
| Fish/seafood-like taste | 7 days  14 days  3 months  6 months | 1 (5.9)  0 (0)  3 (16.7)  3 (16.7) | 0 (0)  0 (0)  3 (16.7)  3 (16.7) | 2 (11.1)  1 (5.6)  8 (44.4)  6 (33.3) | 0 (0)  0 (0)  1 (5.9)  1 (5.9) | > .05  > .05  > .05  > .05 |

*Supplementary Table 2*. Number and percentage of self-perceived adverse events for the Placebo, ATB, IM, and ATB+IM groups at 7 and 14 days (post-ATB/placebo for ATB), and 3 and 6 months (post-IM/placebo for IM) (n=69).

ATB, antibiotics. IM, immunomodulators.

Intergroup comparisons were performed using the Fisher Exact Test with Bonferroni *post-hoc* (p < .05). Subset analysis from the population from Guarulhos University (n=69 patients).
