# Supplementary material for: Immunomodulators, associated or not with systemic antibiotics, to treat periodontitis: A 1‐year multicenter, placebo‐controlled, double‐blind, randomized clinical trial
Source: J Periodontol. 2026 May 12;97(7):1454–66. doi: 10.1002/jper.70081 (PMC13380354; doi:10.1002/jper.70081)
Supplement: Supplementary file 2 — Supporting Information [file JPER-97-1454-s001.docx]

*Supplementary Table 1.* The effect sizes of the ATB, IM, and ATB+IM therapies compared with Placebo, based on the increase in shallow pockets and the reduction in deep pockets from baseline to 1-year post-therapy (n=109 patients).

| Changes in the number of sites between BL-1Y | ATB vs. Placebo  ES (CI 95%) | IM vs. Placebo  ES (CI 95%) | ATB+IM vs. Placebo  ES (CI 95%) |
| --- | --- | --- | --- |
| Δ Sites with PD ≤ 4mm | -0.57 (-1.11, -0.03) – Medium effect | -0.63 (-1.19, -0.07) – Medium effect | -0.50 (-1.04, 0.04) – Medium effect |
| Δ Sites with PD ≥ 5mm | 0.96 (0.40, 1.52) – Large effect | 0.99 (0.41, 1.57) – Large effect | 0.46 (-0.08, 1.01) – Small effect |
| Changes in the number of sites between BL-1Y in patients with ≥ 9 sites with PD ≥ 5 mm | **ATB vs. Placebo**  **ES (CI 95%)** | **IM vs. Placebo**  **ES (CI 95%)** | **ATB+IM vs. Placebo**  **ES (CI 95%)** |
| Δ Sites with PD ≤ 4mm | -0.63 (-1.20, -0.07) – Medium effect | -1.70 (-2.35, -1.04) – Very large effect | -1.30 (-1.91, -0.69) – Very large effect |
| Δ Sites with PD ≥ 5mm | 0.68 (0.12, 1.25) – Medium effect | 0.80 (0.22, 1.38) – Large effect | 0.55 (-0.01, 1.12) – Medium effect |

ATB, antibiotics; CI, confidence interval; ES, effect size; IM, immunomodulators; PD, probing depth.
